# Supplementary material for: An Overview of Off-Label Use of Humanized Monoclonal Antibodies in Paediatrics
Source: Medicina (Kaunas). 2022 Apr 29;58(5):625. doi: 10.3390/medicina58050625 (PMC9144580; doi:10.3390/medicina58050625)
Supplement: Supplementary file 1 [file medicina-58-00625-s001.zip › medicina-1672242-supplementary.pdf]

Supplementary Table S1 -S5

In Supplementary Table S1-S5, we analyze and report the main studies that investigated humanized monoclonal antibodies off-label use in several diseases.

Table S1. Summary of the paediatric case reports on children treated with omalizumab.

Summary of the Paediatric Case Reports on Children Treated with Omalizumab													
CU													
	**Year**	**Study Type**	**Number of Patients**	**Age**	**Sex**	**Comorbidities**	**Total IgE (IU/mL)**	**Previous Treatment**	**Omalizumab Dose (mg)**	**Omalizumab Interval (weeks)**	**Duration of Treatment (months)**	**Response to Treatment**	**Adverse Events**
Viswanathan et al. [1]	2013	Retrospective	1	8	M	NR	270	Cyclosporine	300	4	11	Partial	None
Sussman et al. [2]	2014	Prospective	3	9	M	NR	NR	Antihistamines<br>Montelukast<br>CS<br>Cyclosporine	150	4	NR	Complete	None
				10	M	NR	NR	Antihistamines	300	4	NR	Complete	None
				11	F	NR	NR	Antihistamines<br>Montelukast	150	4	NR	Complete	None
Corral-Magaña et al. [3]	2020	Retrospective, case series	5	2	F	NR	107	Second-generation H1 antihistamines	300	4	12	Complete	Joint pain ($n = 1$)<br>Injection-site reaction ($n = 1$)
				8	F	NR	4590		300	4	6	Partial	
				11	M	NR	209		300	4	6	Complete	
				12	M	NR	905		300	4	3	Complete	
				12	F	NR	65		300	4	12	Complete	
Ari et al. [4]	2020	Retrospective, case series	19	Range: 8–18	11 F<br>8 M	Autoimmune disease ($n = 3$)	NR	NR	300	4	6–24	Complete ($n = 13$)<br>Partial ($n = 3$)<br>Resistant ($n = 3$)	None
Uysal et al. [5]	2014	Case series	2	10	M	NR	94,5	Antihistamines<br>Montelukast<br>CS	150	2	9	Complete	None
				12	F	Delayed pressure Urticaria<br>Urticaria factitia	19,4	Antihistamines	150	2	9	Complete	None
Netchiporou et al. [6]	2015	Case series	3	4	F	NR	919	Antihistamines<br>CS	150	4	6	Complete	None
				5	F	NR	65	Antihistamines<br>CS<br>Montelukast	150	4	6	Complete	None
				10	F	NR	41	Antihistamines<br>Montelukast	300	4	4	Partial	None
										2	2	Complete	None

								CS					
Al-Shaikhly et al. [7]	2019	Case Series	2	11	M	NR	NR	Antihistamines<br>Montelukast	300	4	11	Complete	None
				10.6	M	NR	1147	Antihistamines<br>Montelukast<br>Cyclosporine	300	4	5	Complete	None
Passanisi et al. [8]	2020	Case series	6	15	F	Atopic dermatitis	159	Antihistamines	300	4	6	Complete	None
				16	M	None	388	Antihistamines<br>CS	300	4	6	Complete	None
				16	M	None	112	Antihistamines<br>CS	300	4	6	Complete	None
				16	M	Allergic asthma	110	Antihistamines<br>CS<br>Montelukast	300	4	6	Complete	None
				14	M	Peutz–Jaghers syndrome	21	Antihistamines	300	4	6	No response	None
				11	F	None	163	Antihistamines<br>CS	300	4	6	Complete	None
Asero et al. [9]	2014	Case report	1	11	M	NR	NR	Antihistamines<br>Montelukast<br>CS<br>Cyclosporine	300	4	6	Complete	None
Ossorio-Garcıa et al. [10]	2016	Case report	1	8	F	NR	3281	Antihistamine<br>Cyclosporine<br>Montelukast	300	2	6	Complete	None
Leonardi et al. [11]	2018	Case report	1	7	F	Severe atopic dermatitis	200	Antihistamines	300	4	9	Complete	None
Parisi et al. [12]	2018	Case report	1	12	F	Allergic rhino-conjunctivitis	1685	Antihistamines<br>Montelukast<br>CS	300	4	6	Complete	None
Jesenak et al. [13]	2019	Case report	1	8	M	Diabetes mellitus type 1<br>Autoimmune thyroiditis	Normal value	Desloratadine<br>Montelukast<br>Ranitidine<br>Cyclosporine	300	4	18	Complete	None
Barni et al. [14]	2021	Case report	1	17	F	Crohn's disease	Normal value	Second-generation H1 antihistamines	300	4	2 cycles of 6 months	Complete	None
**Solar urticaria**													
Levi et al. [15]	2015	Case report	1	6	M	Lactose intolerance<br>Attention deficit hyperactivity disorder (ADHD)<br>Allergic rhinitis	2004	Desloratadine<br>Fexofenadine<br>Cetirizine<br>Montelukast	75	2	36	None	None
									150	2		Partial	None

						Mild atopic dermatitis			300	2		Complete	None
Snast et al. [16]	2018	Case report	1	10	F	Asthma<br>Food allergy	243	Antihistamines<br>Montelukast	150	4	17	Complete	None
**Cold-Induced Urticaria**													
Boyce et al. [17]	2006	Case report	1	12	F	Asthma<br>Inhalant allergies	1078	Fluticasone<br>Salmeterol<br>Cetirizine<br>Montelukast	375	2	5	Complete	None
Alba Marín et al. [18]	2015	Case report	1	2	M	NR	14.9	Antihistamines<br>Montelukast	75	4	9	Complete	None
**ASD**													
Jyonouchi H. [19]	2015	Case series	2	12	M	ASD<br>Food protein-induced enterocolitis syndrome (FPIES)<br>Feeding difficulties<br>Chronic sinusitis, allergic rhinitis	NR	Antihistamines<br>Nasal CS<br>Montelukast<br>Topical ophthalmic solutions	300	4 weeks increased to every 2 weeks	NR	Improvement of behavioural symptoms, allergic symptoms	None
				12	M	Delayed-type food allergy<br>Multiple congenital anomalies (bilateral neurosensory hearing loss, agenesis of right kidney and congenital heart)<br>Asthma and seasonal allergy symptoms	NR	Antihistamines<br>Nasal CS Montelukast<br>Topical ophthalmic solutions	NR	NR	NR	Improvement in the cognitive development and allergic symptoms	None
Kong et al. [20]	2021	Case report	1	6	M	ASD<br>Acute disseminated encephalomyelitis<br>Hashimoto's disease<br>Allergic rhinitis and angiedema	695	Antihistamine<br>Nasal CS<br>Montelukast<br>Ranitidine<br>Cromolyn sodium<br>Topic Naltrexon	300 mg every 4 weeks		6	Improvement in allergic symptoms, anxiety, sleep quality, awareness, social communication and cognition	NR
**Allergic Bronchopulmonary Aspergillosis in Cystic Fibrosis Patients**													
Perisson et al. [21]	2017	Retrospective	18	17.1 ±5.2	7 M<br>11 F	NR	3173 kUI/l	CS	300–600	2	6	Stabilization of the lung function<br>CS sparing	None

												Improvement in the nutritional status	
Zirbes et al. [22]	2008	Case series	3	12.9	M	NR	805 KUI/L	CS Itraconazole	300	2	18	Improvement in lung function CS-sparing agent Decrease in exacer-bations	None
				12.8	M	NR	2894 K	CS Itraconazole	375	2	18	Improvement in lung function CS-sparing agent Decrease in exacer-bations	None
				17	M	NR	530 K	CS Itraconazole	300	2	8	Improvement in lung function CS-sparing agent Decrease in exacer-bations	None
Lebecque et al. [23]	2009	Case series	2	14	M	NR	4261	CS Itraconazole	375	2–4	7.5	Improved persistent symptoms and fewer exacerbations	None
				14	F	NR	1526	CS Itraconazole	375	2–4	7.5	Improved persistent symptoms and fewer exacerbations	None
ElMallah et al. [24]	2012	Case series	2	14.5	M	NR	1039	CS	450	4	18	Improved persistent symptoms and fewer exacerbations	None
				11	M	NR	1782	CS	450	2	12	Improved persistent symptoms and fewer exacerbations	None
Wong et al. [25]	2013	Case series	2	14	M	NR	>4000	CS Itraconazole	300	4	24	CS-sparing (CS free for 15 months)	None
				15	M	NR	≅3000	CS Itraconazole	300	4	24	Improvement in lung function CS-sparing (CS free for 17 months)	None
Emiralioglu et al. [26]	2016	Case series	5	14	F	Diabetes mellitus as adverse effect of CS	726	CS Itraconazole	300	4	18	No difference in lung function tests Decrease in respira-tory symptoms and IgE levels CS-sparing agent	None
				16	F	Diabetes mellitus as adverse effect of CS	646	CS Itraconazole	300	4	6		
				11	M	Diabetes mellitus and Cushing syn-drome as adverse ef-fect of CS	725	CS Itraconazole	300	4	18		
				11	F	NR	682	CS Itraconazole	300	4	9		

				13	M	Diabetes mellitus and Cushing syndrome as adverse effect of CS	800	CS<br>Itraconazole	300	4	8	They suggest omalizumab should be started<br>before clinical deterioration and severe decline in lung function tests	
Nové-Josserand et al. [27]	2016	Case series	11	Range: 11–18	3 M<br>8 F	Diabetes mellitus as adverse effect of CS (2/11)	642–5918 (mean 1015)	CS (5/11)<br>Azole therapy (9/11)	150–1200	4	3–50 (mean 18)	CS-sparing (5% discontinue, 50% reduce the dose)<br>No significant difference in lung function or number of intravenous antibiotics	Mild burns at injection site, a skin eruption tingling and swollen lips (4/32)
Parisi et al. [28]	2019	Case series	2	17	M	NR	1124	CS<br>Itraconazole	600	2	NR	Improvement in lung function and symptoms	None
				11	M	NR	1056	CS<br>Antifungal therapy	375	2	NR	Improvement in lung function and symptoms	None
Van der Ent et al. [29]	2007	Case report	1	12	F	NR	5200	CS	300	2	1.5	Improved asthma control and lung function<br>CS-sparing agent	None
Kanu et al. [30]	2008	Case report	1	13	F	NR	947	CS<br>Itraconazole	300	2	3	Improvement in lung function	None
Randhawa et al. [31]	2009	Case report	1	14		CS-induced diabetes	19,506	CS<br>Itraconazole	375	2	11	Diabetes control<br>Maintenance of pulmonary function	None
Brinkmann et al. [32]	2010	Case report	1	15	F	NR	NR	CS<br>Itraconazole<br>Voriconazole	300	4	NR	After initial improvement<br>of lung function, deteriorated again and remained CS dependent	None
						**Food Allergies**							
Fiocchi et al. [33]	2019	Case series	13	Range: 9–18	F 3<br>M 9	Asthma	208–1491 (median 689)	Fluticasone<br>Salmeterol<br>Montelukast	0.016 mg/kg/IgE	2	4	Full tolerance to all foods (n = 9)<br>Full tolerance to at least 1 food (n = 2)<br>Only developed partial tolerance (n = 4)	NR

												Did not achieve any improvement in tolerance (n = 0)	
**High Immunoglobulin E levels in patients with asthma**													
Wang et al. [34]	2018		5 children (11 total patients)	10 (n = 4)<br>11 (n = 1)	2 F<br>3 M	NR	1380<br>1740<br>1830<br>2510<br>4320<br>Mean: 2356<br>Range: 1380–4320	NR	375	2	12	Efficacy of omalizumab in patients with<br>IgE levels higher than the indicated thresholds	Headache and dizziness (1 patient, able to continue therapy)
**Severe Atopic Dermatitis**													
Iyengar et al. [35]	2013	Randomized, placebo-controlled clinical trial	4 (total 8, n = 4 placebo)	Range: 4–22<br>Mean: 11.6	NR	Asthma (6/8) Allergic<br>rhinitis (7/8).	Range: 218–1890 (mean 1068)	NR	150–375	2–4	6	Decreases levels of cytokines (involved in Th2 polarization and allergic inflammation)<br>No differences between 2 groups in clinical outcomes	None
Chan et al. [36]	2020	Randomized clinical trial	30 (total: 62, n = 32 placebo)	10.2	F = 17<br>M = 13	Asthma (n = 11)<br>Food allergies (n = 25)<br>Rhinoconjunctivitis (n = 24)	Range: 4556–18,506 (mean: 8373)	Previous systemic therapy (n = 18)	According to their weight and initial total serum IgE (max 1200 mg/mo)	NR	6	Reduced atopic dermatitis severity and improved quality of life; potent, topical CS-sparing effect	Anaphylaxis (n = 1)<br>Treatment failure (n = 1)
Lane et al. [37]	2006	Case series	3	10	F	Allergy	1990	Topical and oral CS<br>Topical Tacrolimus<br>Montelukast<br>Cyclosporine	300<br>450	2	½<br>continue	Important improvement in skin lesions	None
				13	M	Allergy	6120	Topical and oral CS<br>Topical Tacrolimus<br>Trimethoprim-sulfamethoxazole<br>Montelukast	150<br>300<br>450	2<br>2<br>2	½<br>½<br>continue		

								Cyclosporine					
				12	F	Allergy<br>Asthma	2890	Topic CS<br>Topical Tacroli-mus<br>Montelukast	150<br>300	2<br>2	1<br>continue		
Amrol et al. [38]	2010	Case series	3	12	F	Asthma<br>Allergic rhinitis	36,732	Topical and oral CS<br>Topical Tacroli-mus<br>Montelukast<br>Methotrexate<br>Doxepin	375	2	18	Clinical improvement and decreased exacerba-tions	None
				6	M	Asthma<br>Allergic rhinitis	4090	Topical and oral CS<br>Topical Tacroli-mus<br>Montelukast<br>Doxepin	300	2	3		
García et al. [39]	2019	Case series	6 children ≤ 12 years (19 total pa-tients)	8 (n = 1)<br>9 (n = 3)<br>11(n = 1)<br>10(n = 1)	F = 3<br>M = 3	Asthma (n = 3)<br>Allergic rhinitis (n = 5)<br>Food allergies (n = 2)<br>None (n = 1)	Range: 251 -11.358	Topical CS<br>Topical tacroli-mus<br>Oral CS<br>Athioprine, methotrexate or cyclosporine	150 -600	2–4	Range 6–53 (mean 20.1)	85.7% of patients had an improvement in their quality of life and the severity of the disease	NR
Iannelli et al. [40]	2020	Case series	2	15	NR	Miastenia gravis<br>Asthma	384	CS<br>Pyridostigmine<br>Cyclosporine	600	2	ongoing	Remission of respira-tory symptoms<br>Poor effect on skin lesions	None
				16	NR	Cow's milk allergy<br>Asthma<br>Dust allergy	13,908	Sub-lingual im-munotherapy	450	2	6	Mild benefit on der-matitis<br>After the suspension maintenance, a satis-fying response on both asthma and dermatitis	None
Barni et al. [41]	2017	Case report	1	12	M	Food allergy (egg, milk)<br>Dust allergy	750	Topical and oral CS<br>Topical Tacroli-mus<br>Cyclosporine<br>Methotrexate	300	4	16	Excellent clinical re-sponse (50% SCORAD reduction)<br>Improved quality of life	None
							**Chronic Rhinosinusitis and Nasal Polyposis**						

Shoda et al. [42]	2019	Case report	1	8	M	Asthma Allergic rhinitis	151	Montelukast Inhaled and systemic CS Cyclosporine	150- 300	4	30	Effective for asthma Insufficient effect on sinusitis	None
**Vernal Keratoconjunctivitis**													
Heffler et al. [43]	2016	Case series	1 (2 total)	7	F	Eczema	NR	Antihistamines Mast cell stabilizer CS Cyclosporine Tacrolimus	600	4	6	Complete	None
Occasi et al. [44]	2017	Case series	4	6	M	Eczema	NR	Topical antihistamines Topical Cyclosporine Topical Tacrolimus	225	2	6	Improvement of ocular symptoms No relapse after treatment suspension	NR
				8	M	None	NR		300	4			
				11	F	Persistent rhinitis	NR		225	4			
				9	M	Persistent rhinitis	NR		225	4			
Doan et al. [45]	2016	Case series	4	13	M	Asthma Rhinitis	146	Topical CS Topical Cyclosporine	600	2	16	Partial control	None
				10	M	Asthma Rhinitis	1655	Topical CS Topical Cyclosporine CS subtarsal injection	600	2	33 (still ongoing)		
				7	M	Asthma Eczema Rhinitis	800	Topical CS Topical Cyclosporine	450	2	42 (still ongoing)		
				7	M	Asthma	141	Topical CS Topical Cyclosporine CS subtarsal injection	600	2	6	Failure	None
Manti et al. [46]	2021	Case series, review	2	12	M	Asthma	2004	Topical antihistamines Topical mast cell stabilizers Topical CS Topical Cyclosporine	450	2	9	Improvement in clinical symptoms, in severity disease, in need for rescue therapies and in ocular examination findings	None
				10	M	Asthma	2498		600	2	9		
Sánchez et al.[47]	2011	Case report	1	15	M	Asthma Atopic dermatitis Rhinitis	340	Topical Cyclosporine Topical CS	300	2	9	Important improvement in ocular symptoms	None

								Topical Tacrolimus					
De Klerk et al. [48]	2012	Case report	1	12	M	Asthma<br>Eczema<br>Rhinitis	NR	Topical antihistamines<br>Topical mast cell stabilizer<br>CS subtarsal injection and topical<br>Topical Cyclosporine	300	4	18	Marked improvement<br>in ocular symptoms	None

Table S2. Summary of the paediatric case reports on children treated with mepolizumab.

						Mepolizumab							
	**Year‡**	**Study type**	**Number of Patients**	**Age**	**Sex**	**Comorbidities**	**Blood Eosinophilia**	**Previous Treatment**	**Mepolizumab Dose**	**Mepolizumab Interval (Weeks)**	**Duration of Treatment**	**Response to Treatment**	**Adverse Reaction**
						**Eosinophilic esophagitis**							
Ass'ad et al. [49]	2010	International, multicenter, double-blind, randomized, stratified, parallel-group study	59	2–17 (mean 10.4)	F = 12<br>M = 37	NR	NR	CS	0.55 mg/kg (n = 19)<br>2.5 mg/kg (n = 20)<br>10 mg/kg (n = 20)	4	12 weeks	Complete response<br>was only achieved in 8.8%<br>Reduction in esophageal<br>mast cell accumulation and numbers of intraepithelial eosinophils	86.4% reported ≥1 adverse events; the most frequent were gastrointestinal (vomiting (16.9%), diarrhea (13.6%) and upper abdominal pain (10.2%))<br>No dose-related trends in adverse event rates<br>No hypersensitivity reactions.<br>3 serious adverse events (chest pain, food stuck in throat and esophageal injury secondary to endoscopy)
Otani et al. [50]	2013	Case series	43	NR	NR	NR	NR	NR	0.55 mg/kg<br>2.5 mg/kg<br>10 mg/kg	4	12 weeks	Reduction in esophageal<br>mast cell accumulation (77% of all subjects) and numbers of intraepithelial eosinophils	NR

												40% of subjects responded.	
							**HES**						
Mehr et al. [51]	2009	Case report	1	12	M	Asthma	19,700,000,000/L	CS<br>Interferon alpha<br>Imatinib mesylate	10 mg/kg	4	Maintenance regimen every 3 months	Better control of disease flares<br>Maintenance blood eosinopenia<br>CS-sparing	None
Schwarz et al. [52]	2018	Case series	1	14	M	Asthma	45%	Azathioprine<br>Imatinib	750 mg	4–6	4 years	Very good efficacy	None
			1	11	F	NR	NR	CS<br>β2-adrenergic agonists<br>Azathioprine<br>Methotrexate	750 mg	4–6	7 years	CS-sparing<br>Good control of eosinophilia	None
Domany et al. [53]	2020	Case report	1	8	M	Asthma	40.000/μL	systemic CS<br>Hydroxyurea	NR	NR	NR	CS-sparing clearing eosinophil lung infiltration<br>Partial improvement in asthma control	NR
							**Aspirin-Exacerbated Respiratory Disease**						
Méndez Sánchez et al. [54]	2018	Case report	1	12	M	Asthma, chronic rhinosinusitis and nasal polyps	24.3%	CS<br>Antihistamines<br>Montelukast<br>long-acting β-2-agonist	NR	4	1 year	Significant clinical and functional improvement with good asthma control	NR
							**Refractory thoracic conidiobolomycosis**						
Yeoh et al.[55]	2021	Case report	1	6	M	NR	790 cells/mL	Miltefosine<br>Terbinafine<br>Amphotericin B<br>CS	5 mg/kg	4	1 year	Complete.<br>CS-sparing agent<br>At the 12-month follow-up no clinical, hematological, biochemical or radiological evidence of relapse	NR

Table S3. Summary of the paediatric case reports on children treated with dupilumab.

							**Dupilumab**						
	**Year**	**Study Type**	**Number of Patients**	**Age**	**Sex**	**Comorbidities**	**Previous Treatment**	**Concomitant Treatment**	**Dupilumab Dose**	**Interval (Weeks)**	**Duration of Treatment (Months)**	**Response to Treatment**	**Adverse Events**

**Alopecia areata**													
Penzi et al. [56]	2018	Case report	1	13	F	Treatment-resistant AD	Topical squaric acid and nthralin<br>Prednisone<br>Methotrexate	None	300	NR	11	Improvement in AD and in hair growth	Minimal dry eye
Gruenstein et al. [57]	2020	Case report	1	4	F	Treatment-resistant AD	NR	None	200	2	4	AD well controlled<br>Scalp hair has fully regrown	NR
Cho et al. [58]	2021	Case series	6	12	M	Asthma<br>Food allergies	Topical CS<br>Pimecrolimus<br>Tacrolimus	None	NR	NR	24	73% improvement in the severity of alopecia tool score	None
				7	M	Asthma	Topical CS<br>Tacrolimus<br>Crisaborole	None			6	Complete regrowth	None
				7	F	Food allergies	Topical CS<br>Tacrolimus	Pulsed prednisone 5 mg/kg monthly x 6 doses,<br>Oral Minoxidil			16	Complete regrowth	Mild, transient conjunctivitis
				8	F	None	Topical CS<br>Tacrolimus<br>Crisaborole	Oral Minoxidil<br>Topical Tofacitinib			6	Complete regrowth	None
				7	F	None	Topical CS<br>Pimecrolimus	Topical Tofacitinib<br>Topical Minoxidil			6		None
				12	F	Food allergies	Topical CS<br>Tacrolimus	Oral inoxidil<br>Topical CS			16	No response	None
Mckenzie et al. [59]	2021	Case series	16	Range: 8–19	10 F<br>6 M	AD (16/16)<br>Asthma (4/16)	Oral CS (12/16)<br>Methotrexate (9/12)	Topical tofacitinib (2/16)<br>Oral methotrexate (1/16)<br>Oral tofacinitib (2/16)<br>Intralesional CS (2/16)<br>Minoxidil (2/16)<br>Spiromolactone (1/16)	300	2	2–31	Clinical improvement of their AD and asthma<br>Worsening in severity (4/12), improved in time<br>No/minimal improvement (2/12)	Mild injection site reactions (3/12)
**Dyshidrotic eczema**													

Weins et al. [60]	2019	Case report	1	12	M	AD in early childhood	Topical and systemic Antimicrobials, as well as UVA<br>Topical CS<br>Oral CS<br>Methotrexate<br>cyclosporine A	Topical and oral CS	600 day 0<br>300	2	4	Complete stable remission	None
**Eosinophilic annular erythema**													
Gordon et al. [61]	2018	Case report	1	14	F	NR	Topical and oral CS<br>Oral dapsone<br>Oral tofacitinib	None	600 day 0<br>300	2	5- ongoing	Resolution of all lesions and associated pruritus	None
**Prurigo nodularis**													
Fachler et al. [62]	2020	Case report	1	9	F	NR	Topical CS<br>Antihistamines<br>Ultraviolet B phototherapy<br>Cyclosporine<br>Intralesional CS<br>Oral methotrexate	NR	200 day 0<br>100	2	12	Complete regression of lesions<br>Reduction in pruritus	None
**Actinic Prurigo**													
Eickstaedt et al. [63]	2020	Case report	1	7	F	NR	Topical CS<br>Antihistamines<br>Cyclosporine<br>Methotrexate	Methotrexate (2 months overlap)	400 day 0<br>200	2	7	Regression of lesions<br>Methotrexate interruption	Mild ocular pruritus<br>Transient injection site reaction
**ANCA-negative eosinophilic granulomatosis with polyangiitis**													
Galant-Swafford et al. [64]	2020	Case series	2	14	M	Asthma, allergic rhinitis, CRSwNP	Inhaled and oral CS<br>Montelukast<br>Omalizumab<br>Mepolizumab<br>Rituximab	Mepolizumab (1 month overlap)	600 day 0<br>300	2	7	CS-sparing agent<br>Improvement in skin disease and lung function	None
				16	F	CRSwNP	Inhaled and oral CS<br>Methotrexate<br>Adalimumab<br>Rituximab<br>Mepolizumab	Mepolizumab (2 months overlap)	600 day 0<br>300	2			

Table S4. Summary of the paediatric case reports on children treated with reslizumab.

Reslizumab													
**Eosinophilic Esophagitis**													
	**Year**	**Study Type**	**Number of Patients**	**Number of Patients in Each Subgroup**	**Age, mean (SD)**	**Sex**	**Comorbidities**	**Previous Treatment**	**Reslizumab Dose (mg/kg)**	**Reslizumab Interval (Weeks)**	**Duration of Treatment**	**Response to treatment**	**Adverse Reaction**
Spergel et al. [65]	2011	Double-blind, randomized, placebo-controlled trial	227	55	12.3 (3.83)	41 M 14 F	Asthma (28) Chronic sinusitis (10) Nasal polyps (0) Atopic dermatitis (21) Allergic rhinitis (38)	NR	1	Infusions of 1, 2, or 3 mg/kg reslizumab or placebo at weeks 0, 4, 8, and 12.	3 months	Median reductions from baseline in peak esophageal eosinophil counts were 59%, 67%, 64% and 24% in the 1, 2 and 3 mg/kg reslizumab (all $p < 0.001$) and placebo groups, respectively All treatment groups, including the placebo group, showed improvements in physician's global assessment score The differences between the reslizumab and placebo groups were not statistically significant	The most common adverse events in the reslizumab groups were headache, cough, nasal congestion and upper respiratory tract infection $n = 1$ in each reslizumab group and $n = 2$ in the placebo group had serious adverse events; none was considered related to the study medication
				57	11.8 (3.82)	42 M 15 F	Asthma (24) Chronic sinusitis (5) Nasal polyps (1) Atopic dermatitis (21) Allergic rhinitis (33)		2				
				57	11.5 (4.04)	45 M 12 F	Asthma (32) Chronic sinusitis (10) Nasal polyps (0) Atopic dermatitis (26) Allergic rhinitis (35)		3				
				57	11.9 (4.17)	44 M 13 F	Asthma (25) Chronic sinusitis (8) Nasal polyps (2) Atopic dermatitis (23)		Placebo				

Markowitz et al. [66]	2018	Multicenter, randomized, control trial study	12	-	12 (4)	9 M<br>3 F	Allergic rhinitis (33)<br>NR	CS (4/12)<br>Diet restriction (10/12)<br>Elemental diet (1/12)<br>PPI (12/12)	1–3	4	From 6 months to 4 years	Reslizumab appears to be safe over 9 years of treatment experience Symptoms and eosinophil count improved considerably during treatment	Cough and nasal congestion (4/12)<br>Upper respiratory tract infection (2/12)<br>Otitis media (2/12)<br>Constipation (1/12)<br>Headache (2/12)<br>Toothache (1/12)<br>Back pain (1/12)<br>Pain on infusion arm (2/12)<br>No serious adverse event

Table S5. Summary of the paediatric case reports on children treated with benralizumab.

**Benralizumab**														
**Pulmonary eosinophilia**														
	**Year**	**Study Type**	**Number of Patients**	**Age**	**Sex**	**Comorbidities**	**IgE**	**Previous Treatment**	**Concomitant Treatment**	**Benralizumab Dose (mg)**	**Benralizumab Interval (weeks)**	**Duration of Treatment (months)**	**Response to Treatment**	**Adverse Reaction**
Hinds et al. [67]	2021	Case Series	3	14	F	Asthma<br>Pulmonary hypertension<br>Factor VIII elevation with venous thromboses ABPA	5988 kUA/L	CS (Methylprednisolone)	Rituximab	30<br>30	4<br>8	3<br>3	CS-sparing agent	None
				7	F	Asthma	Normal	CS (prednisolone)	CS	30<br>30	4<br>8	3<br>4	Symptoms improved immediately<br>FEV1% improvement<br>CS-sparing agent	None
				15	F		Normal	CS (Methylprednisolone)	CS	30<br>30	4<br>8	3<br>3	Resolution of bronchial	None

													dilation FEV1% improvement CS-sparing agent	
							HES							
Forero-Molina et al. [68]	2020	Case report	1	9	F	Severe eczema	NR	CS Imatinib	CS	30	NR	18	CS-sparing agent End-organ damage, including heart failure and Arrhythmia, successfully treated	None

References

1. Viswanathan, R.K.; Moss, M.H.; Mathur, S.K. Retrospective analysis of the efficacy of omalizumab in chronic refractory urticaria. *Allergy Asthma Proc.* **2013**, *34*, 446–452.
2. Sussman, G.; Hébert, J.; Barron, C.; Bian, J.; La, S.; Stern, S. Real-life experiences with omalizumab for the treatment of chronic urticaria. *Ann. Allergy Asthma Immunol.* **2014**, *112*, 170–174.
3. Corral-Magaña, O.; Gil-Sánchez, J.A.; Bover-Bauzá, C.; Martinez-Cepas, P.; Montis-Palos, M.C.; Martín-Santiago, A. Chronic urticaria in children under 15 years of age: Clinical experience beyond the clinical trials. *Pediatr. Dermatol.* **2021**, *38*, 385–389.
4. Ari, A.; Levy, Y.; Maoz-segal, N.S.R.; Benor, S.; Broides, A.; Horev, A.; Epstein-rigbi, N.; Agmon-levin, N.; Marcus, N.; Tikva, P. Efficacy of omalizumab treatment for paediatric chronic spontaneous urticaria : A multi-center retrospective case series. *Pediatr. Dermatol.* **2020**, *37*, 1051–1054.
5. Uysal, P.; Eller, E.; Mortz, C.G.; Bindslev-Jensen, C. An algorithm for treating chronic urticaria with omalizumab: Dose interval should be individualized. *J. Allergy Clin. Immunol.* **2014**, *133*, 914–915.
6. Netchiporouk, E.; Nguyen, C.H.; Thuraisingham, T.; Jafarian, F.; Maurer, M.; Ben-Shoshan, M. Management of pediatric chronic spontaneous and physical urticaria patients with omalizumab: Case series. *Pediatr. Allergy Immunol.* **2015**, *26*, 585–588.
7. Al-Shaikhly, T.; Rosenthal, J.A.; Ayars, A.G.; Petroni, D.H. Omalizumab for chronic urticaria in children younger than 12 years. *Ann. Allergy Asthma Immunol.* **2019**, *123*, 208–210.
8. Passanisi, S.; Arasi, S.; Caminiti, L.; Crisafulli, G.; Salzano, G.; Pajno, G.B. Omalizumab in children and adolescents with chronic spontaneous urticaria: Case series and review of the literature. *Dermatol. Ther.* **2020**, *33*, e13489.
9. Asero, R.; Casalone, R.; Iemoli, E. Extraordinary response to omalizumab in a child with severe chronic urticaria. *Eur. Ann. Allergy Clin. Immunol.* **2014**, *46*, 41–42.
10. Ossorio-García, L.; Jiménez-Gallo, D.; Albarrán-Planelles, C.; Arjona-Aguilera, C.; Linares-Barrios, M. Chronic spontaneous urticaria in an 8-year-old girl treated with omalizumab. *Clin. Exp. Dermatol.* **2016**, *41*, 929–930.
11. Leonardi, L.; Uva, A.; Duse, M. Chronic urticaria in a child affected by atopic dermatitis: Effective treatment with omalizumab. *J. Dermatolog. Treat.* **2018**, *29*, 17–19.
12. Parisi, G.F.; Papale, M.; Tardino, L.G.; Manti, S.; Cuppari, C.; Salpietro, C.; Leonardi, S. Omalizumab treatment in a 12 year-old girl with chronic spontaneous urticaria Giuseppe. *J. Dermatolog. Treat.* **2018**, *29* (Suppl. 4), 10–11.
13. Jesenak, M.; Ciljakova, M.; Janickova, M.; Banovcin, P. Omalizumab in an 8-year-old boy with diabetes mellitus and refractory chronic spontaneous urticaria. *J. Investig. Allergol. Clin. Immunol.* **2019**, *29*, 144–146.

14. Barni, S.; Giovannini, M.; Liccioli, G.; Sarti, L.; Gissi, A.; Lionetti, P.; Mori, F. Case Report: Refractory Chronic Spontaneous Urticaria Treated With Omalizumab in an Adolescent With Crohn's Disease. *Front. Immunol.* **2021**, *12*, 1–5.
15. Levi, A.; Tal, Y.; Dranitzki, Z.; Shalit, M.; Enk, C.D. Successful omalizumab treatment of severe solar urticaria in a 6-year-old child. *Pediatr. Allergy Immunol.* **2015**, *26*, 588–590.
16. Snast, I.; Kremer, N.; Lapidoth, M.; Enk, C.D.; Tal, Y.; Rosman, Y.; Confino-cohen, R.; Hodak, E.; Levi, A. Omalizumab for the treatment of solar urticaria: Case series and systematic review of the literature. *J. Allergy Clin. Immunol. Pract.* **2018**, *6*, 1198–1204.
17. Boyce, J.A. Successful treatment of cold-induced urticaria/anaphylaxis with anti-IgE. *J. Allergy Clin. Immunol.* **2006**, *117*, 1415–1418.
18. Alba Marín, J.C.; Martorell Aragones, A.; Satorre Viejo, I.; Gastaldo Simeon, E. Treatment of severe cold-induced urticaria in a child with omalizumab. *J. Investig. Allergol. Clin. Immunol.* **2015**, *25*, 303–304.
19. Jyonouchi, H. Marked improvement of neuropsychiatric symptoms following control of allergy symptoms with the use of humanized murine anti IgE antibody (omalizumab) in 2 patients with severely limited expressive language. *Allergy Asthma Clin. Immunol.* **2015**, *11*, 1–4.
20. Kong, X.J.; Clairmont, C.; Wang, B. Case Report: Off-Label Use of Omalizumab in a 6-Year-Old Boy With ASD Ameliorated Severe Allergic Rhinitis and Subsequently Improved Behavioral Symptoms. *Front. Pediatr.* **2021**, *9*, 1–7.
21. Perisson, C.; Destruys, L.; Grenet, D.; Bassinet, L.; Derelle, J.; Sermet-Gaudelus, I.; Thumerelle, C.; Prevotat, A.; Rosner, V.; Clement, A.; et al. Omalizumab treatment for allergic bronchopulmonary aspergillosis in young patients with cystic fibrosis. *Respir. Med.* **2017**, *133*, 12–15.
22. Zirbes, J.M.; Milla, C.E. Steroid-sparing effect of omalizumab for allergic bronchopulmonary aspergillosis and cystic fibrosis. *Pediatr. Pulmonol.* **2008**, *43*, 607–610.
23. Lebecque, P.; Leonard, A.; Argaz, M.; Godding, V.; Pilette, C. Omalizumab for exacerbations of allergic bronchopulmonary aspergillosis in patients with cystic fibrosis. *BMJ Case Rep.* **2009**, *2009*:bcr07.2008.0379.
24. Elmallah, M.K.; Hendeles, L.; Hamilton, R.G.; Capen, C.; Schuler, P.M. Management of patients with cystic fibrosis and allergic bronchopulmonary aspergillosis using anti-immunoglobulin e therapy (omalizumab). *J Pediatr Pharmacol Ther.* **2012**, *17*, 88–92.
25. Wong, R.; Wong, M.; Robinson, P.D.; Fitzgerald, D.A. Omalizumab in the management of steroid dependent Allergic Bronchopulmonary Aspergillosis (ABPA) complicating Cystic Fibrosis. *Paediatr. Respir. Rev.* **2013**, *14*, 22–24.
26. Emiralioglu, N.; Dogru, D.; Tugcu, G.D.; Yalcin, E.; Kiper, N.; Ozcelik, U. Omalizumab Treatment for Allergic Bronchopulmonary Aspergillosis in Cystic Fibrosis. *Ann. Pharmacother.* **2016**, *50*, 188–193.
27. Nové-Josserand, R.; Grard, S.; Auzou, L.; Reix, P.; Murris-Espin, M.; Brémont, F.; Mammar, B.; Mely, L.; Hubert, D.; Durieu, I.; et al. Case series of omalizumab for allergic bronchopulmonary aspergillosis in cystic fibrosis patients. *Pediatr. Pulmonol.* **2017**, *52*, 190–197.
28. Parisi, G.F.; Portale, A.; Papale, M.; Tardino, L.; Rotolo, N.; Licari, A.; Leonardi, S. Successful treatment with omalizumab of allergic bronchopulmonary aspergillosis in patients with cystic fibrosis: Case reports and literature review. *J. Allergy Clin. Immunol. Pract.* **2019**, *7*, 1636–1638.
29. Van Der Ent, C.K.; Hoekstra, H.; Rijkers, G.T. Successful treatment of allergic bronchopulmonary aspergillosis with recombinant anti-IgE antibody. *Thorax* **2007**, *62*, 276–277.
30. Kanu, A.; Patel, K. Treatment of allergic bronchopulmonary aspergillosis (ABPA) in CF with Anti-IgE antibody (Omalizumab). *Pediatr. Pulmonol.* **2008**, *43*, 1249–1251.
31. Randhawa, I.; Chin, T.; Nussbaum, E. Resolution of corticosteroid-induced diabetes in allergic bronchopulmonary aspergillosis with omalizumab therapy: A novel approach. *J. Asthma* **2009**, *46*, 445–447.
32. Brinkmann, F.; Schwerk, N.; Hansen, G.; Ballmann, M. Steroid dependecy despite omalizumab treatment of ABPA in cystic fibrosis. *Allergy Eur. J. Allergy Clin. Immunol.* **2010**, *65*, 134–135.
33. Fiocchi, A.; Artesani, M.C.; Riccardi, C.; Mennini, M.; Pecora, V.; Fierro, V.; Calandrelli, V.; Dahdah, L.; Valluzzi, R.L. Impact of Omalizumab on Food Allergy in Patients Treated for Asthma: A Real-Life Study. *J. Allergy Clin. Immunol. Pract.* **2019**, *7*, 1901–1909.
34. Wang, K.Y.; Sindher, S.B.; Stinson, R.; DaVeiga, S.P. Efficacy and safety of omalizumab in paediatric patients with high immunoglobulin E levels: A case series. *Allergy Asthma Proc.* **2018**, *39*, 289–291.
35. Yengar, S.R.; Hoyte, E.G.; Loza, A.; Bonaccorso, S.; Chiang, D.; Umetsu, D.T.; Nadeau, K.C. Immunologic Effects of Omalizumab in Children with Severe Refractory Atopic Dermatitis : A Randomized , Placebo-Controlled Clinical Trial. *Int. Arch. Allergy Immunol.* **2013**, *162*, 89–93.
36. Chan, S.; Cornelius, V.; Cro, S.; Harper, J.I.; Lack, G. Treatment Effect of Omalizumab on Severe Paediatric Atopic Dermatitis: The ADAPT Randomized Clinical Trial. *JAMA Pediatr.* **2020**, *174*, 29–37.
37. Lane, J.E.; Cheyney, J.M.; Lane, T.N.; Kent, D.E.; Cohen, D.J. Treatment of recalcitrant atopic dermatitis with omalizumab. *J. Am. Acad. Dermatol.* **2006**, *54*, 68–72.

38. Amrol, D. Anti-Immunoglobulin E in the Treatment of Refractory Atopic Dermatitis. *South Med. J.* **2010**, *103*, 554–558.
39. García, M.; Durán-Crane, A.; Chapman, E.; García, E. Omalizumab as an adjuvant therapy for treating severe atopic dermatitis in children. A serie of cases. *Rev. Alerg. Mex.* **2019**, *66*, 282–291.
40. Iannelli, M.; Caminiti, L.; Vaccaro, M.; Marafioti, I.; Spinuzza, A.; Panasiti, I.; Barbalace, A.; Crisafulli, G.; Pajno, G.B. Omalizumab for treatment of refractory severe atopic dermatitis. A paediatric perspective. *Dermatol. Ther.* **2020**, *33*, e13519.
41. Barni, S.; Mori, F.; Bortone, B.; Novembre, E. Personalized treatment of severe atopic dermatitis in a child: A ten-year follow-up. *Pediatr. Allergy Immunol.* **2017**, *28*, 701–703.
42. Shoda, Y.; Watanabe, M.; Wada, K.; Soutome, T.; Komine, Y.; Mikami, T.; Nemoto, T.; Ohara, A. Successful management of severe asthma in a young boy with eosinophilic chronic rhinosinusitis who received omalizumab: A case report. *Allergy Asthma Clin. Immunol.* **2019**, *15*, 1–6.
43. Heffler, E.; Picardi, G.; Liuzzo, M.T.; Pistorio, M.P.; Crimi, N. Omalizumab treatment of vernal keratoconjunctivitis. *JAMA Ophthalmol.* **2016**, *134*, 461–463.
44. Occasi, F.; Duse, M.; Nebbioso, M.; De Castro, G.; Di Fraia, M.; Capata, G.; Lollobrigida, V.; Zicari, A.M. Vernal keratoconjunctivitis treated with omalizumab: A case series. *Pediatr. Allergy Immunol.* **2017**, *28*, 503–505.
45. Doan, S.; Amat, F.; Gabison, E.; Saf, S.; Cochereau, I.; Just, J. Omalizumab in Severe Refractory Vernal Keratoconjunctivitis in Children : Case Series and Review of the Literature. *Ophthalmol. Ther.* **2017**, *6*, 195–206.
46. Manti, S.; Parisi, G.F.; Papale, M.; Marseglia, G.L.; Licari, A.; Leonardi, S. Clinical efficacy and safety of omalizumab in conventional treatment-resistant vernal keratoconjunctivitis : Our experience and literature review. *Immun. Inflamm. Dis.* **2021**, *9*, 3–7.
47. Sánchez, J.; Cardona, R. Omalizumab. An option in vernal keratoconjunctivitis ? *Allergol. Immunopathol.* **2012**, *40*, 319–320.
48. De Klerk, T.A.; Sharma, V.; Arkwright, P.D.; Biswas, S. Severe vernal keratoconjunctivitis successfully treated with subcutaneous omalizumab. *J. AAPOS* **2013**, *17*, 305–306.
49. Assa'ad, A.H.; Gupta, S.K.; Collins, M.H.; Thomson, M.; Heath, A.T.; Smith, D.A.; Perschy, T.L.; Jurgensen, C.H.; Ortega, H.G.; Aceves, S.S. An Antibody Against IL-5 Reduces Numbers of Esophageal Intraepithelial Eosinophils in Children With Eosinophilic Esophagitis. *Gastroenterology* **2011**, *141*, 1593–1604.
50. Otani, I.M.; Anilkumar, A.A.; Newbury, R.O.; Bhagat, M.; Beppu, L.Y.; Dohil, R.; Broide, D.H.; Aceves, S.S. Anti-IL-5 Therapy Reduces Mast Cells and IL-9 Cells in Paediatric Eosinophilic Esophagitis. *J. Allergy Clin. Immunol.* **2013**, *131*, 1576–1582.
51. Mehr, S.; Rego, S.; Kakakios, A.; Kilham, H.; Kemp, A. Treatment of a Case of Paediatric Hypereosinophilic Syndrome with Anti-Interleukin-5. *J. Pediatr.* **2009**, *155*, 289–291.
52. Schwarz, C.; Müller, T.; Lau, S.; Parasher, K.; Staab, D. Mepolizumab—A novel option for the treatment of hypereosinophilic syndrome in childhood. *Pediatric Allergy Immunol.* **2018**, *29*, 28–33.
53. Armoni Domany, K.; Shiran, S.I.; Adir, D.; Lavie, M.; Levin, D.; Diamant, N.; Gut, G.; Benor, S. The Effect of Mepolizumab on the Lungs in a Boy with Hypereosinophilic Syndrome. *Am. J. Respir. Crit. Care Med.* **2020**, *202*, 34–35.
54. Martínez JR, G.; Piñera, V. Aspirin-Exacerbated Respiratory Disease in a Paediatric Patient Treated with Mepolizumab. *Arch. Bronconeumol.* **2019**, *55*, 55–57.
55. Yeoh, D.K.; Saunders, T.; Butters, C.; Burgner, D.; Bryant, P.A.; Cain, T.M.; Ng, J.; Gwee, A.; Daley, A.J.; Cole, T.; et al. Refractory thoracic conidiobolomycosis treated with mepolizumab immunotherapy. *J. Allergy Clin. Immunol. Pract.* **2021** *9*, 2527–2530.
56. Penzi, L.R.; Yasuda, M.; Manatis-Lornell, A.; Hagigeorges, D. Hair Regrowth in a Patient With Long-standing Alopecia Totalis and Atopic Dermatitis Treated With Dupilumab. *JAMA Dermatol.* **2018**, *202*, e34–e35.
57. Gruenstein, D.; Malik, K.; Levitt, J. Full scalp hair regrowth in a 4-year-old girl with alopecia areata and atopic dermatitis treated with dupilumab. *JAAD Case Rep.* **2020**, *6*, 1286–1287.
58. Cho, S.K.; Craiglow, B.G. Dupilumab for the treatment of alopecia areata in children with atopic dermatitis. *JAAD Case Rep.* **2021**, *16*, 82–85.
59. McKenzie, P.L.; Castelo-Soccio, L. Dupilumab therapy for alopecia areata in paediatric patients with concomitant atopic dermatitis. *J. Am. Acad. Dermatol.* **2020**, *84*, 1691–1694.
60. Weins, A.B.; Biedermann, T.; Eyerich, K.; Moeckel, S.; Schnopp, C. Successful treatment of recalcitrant dyshidrotic eczema with dupilumab in a child. *J. Dtsch. Dermatol. Ges.* **2019**, *17*, 1165–1167.
61. Gordon, S.C.; Robinson, S.N.; Abudu, M.; Her, M.; Deverapalli, S.; Levin, A.; Schmidt, B.A.; Gellis, S.E.; Rosmarin, D. Eosinophilic annular erythema treated with dupilumab. *Pediatr. Dermatol.* **2018**, *35*, e255–e256.
62. Fachler, T.; Maria Faitataziadou, S.; Molho-Pessach, V. Dupilumab for paediatric prurigo nodularis : A case report. *Pediatr. Dermatol.* **2021**, *38*, 334–335.
63. Eickstaedt, J.B.; Starke, S.; Krakora, D.; Hinshaw, M.; Arkin, L.M. Clearance of paediatric actinic prurigo with dupilumab. *Pediatr. Dermatol.* **2020**, *37*, 1176–1178.

64. Galant-Swafford, J.; Geng, B.; Leibel, S.; Akuthota, P.; Tucker, S.; Cernelc-Kohan, M.; Sheets, R.; Nation, J.; Jefferson, A.A. Two paediatric cases of ANCA-negative eosinophilic granulomatosis with polyangiitis successfully treated with dupilumab. *J. Allergy Clin. Immunol. Pract.* **2020**, *8*, 3643–3646.
65. Spergel, J.M.; Rothenberg, M.E.; Collins, M.H.; Furuta, G.T.; Markowitz, J.E.; Fuchs, G.; O'Gorman, M.A.; Abonia, J.P.; Young, J.; Henkel, T.; et al. Reslizumab in children and adolescents with eosinophilic esophagitis: Results of a double-blind, randomized, placebo-controlled trial. *J. Allergy Clin. Immunol.* **2012**, *129*, 456–463.
66. Markowitz, J.E.; Jobe, L.; Miller, M.; Frost, C.; Laney, Z.; Eke, R. Safety and Efficacy of Reslizumab for Children and Adolescents With Eosinophilic Esophagitis Treated for 9 Years. *Pediatr. Gastroenterol. Nutr.* **2018**, *66*, 893–897.
67. Hinds, D.M.; Bloom, J.L.; Cooper, J.C.; Dutmer, C.M.; Galambos, C.; Weinman, J.P.; Wechsler, M.E.; Liptzin, D.R. Pulmonary eosinophilic vasculitis with granulomas and benralizumab in children. *Pediatr. Pulmonol.* **2021**, *56*, 1789–1792.
68. Molina, M.A.F.; Coffey, K.E.; Chong, H.J. Successful treatment of idiopathic hypereosinophilic syndrome with benralizumab in a paediatric patient. *J. Allergy Clin. Immunol. Pract.* **2021**, *9*, 589–590.
